# Counteracting microgravity: preserving cardiovascular health in low earth orbit

**DOI:** 10.1186/s40779-025-00642-y

**Published:** 2025-09-01

**Authors:** Alan Silburn

**Affiliations:** University of Tasmania, Hobart, TAS 7001 Australia

**Keywords:** Microgravity, Cardiovascular health, Low earth orbit, Gravity Loading Countermeasure Skinsuit, Spaceflight exercise, Cardiac muscle atrophy

Dear Editor,

As space exploration transitions from short orbital missions to extended stays on the International Space Station (ISS) and, ultimately, interplanetary travel, astronaut health has emerged as a critical focus. In particular, safeguarding cardiovascular function has become an operational imperative. Yet beyond safeguarding those in orbit, the physiological adaptations observed in microgravity offer a compelling lens through which to examine persistent challenges in terrestrial medicine, from orthostatic intolerance in the elderly to deconditioning in critical care survivors. By studying how the human cardiovascular system functions in the absence of gravity, we can gain valuable insights and strategies for managing circulatory and autonomic dysfunction here on Earth.

On Earth, the cardiovascular system is constantly working against gravity to maintain blood flow to vital organs. When an individual moves from lying down to standing, approximately 500 ml of blood shifts to the lower extremities. This triggers the arterial baroreflex, a reflex arc that detects changes in blood pressure through baroreceptors in the carotid sinus and aortic arch. These receptors initiate a cascade via the autonomic nervous system to increase heart rate, vascular tone, and cardiac contractility, thus maintaining cerebral perfusion [1, 2]. This system is finely balanced and essential to daily function. In microgravity, however, this dynamic is fundamentally altered.

Without gravitational pull, the body’s fluid distribution becomes uniform, causing a cephalad shift in blood volume and a reduction in plasma volume by 10–15% [3]. Central venous pressure falls, and the stimuli required to maintain baroreflex tone are diminished. Over time, this leads to deconditioning of the baroreflex itself. Astronauts returning from extended missions often experience symptoms such as dizziness, postural tachycardia, and even syncope, clinically related to postural orthostatic tachycardia syndrome or orthostatic hypotension [4]. The parallels to conditions affecting aging adults and patients recovering from prolonged immobilisation are striking.

Compounding these regulatory changes is the issue of cardiac atrophy. Like skeletal muscle, cardiac tissue remodels in response to mechanical load. Terrestrially, physical activity and gravitational resistance maintain myocardial mass and strength. In space, the heart is effectively deconditioned. Long-duration studies, including those from skylab and the ISS, have shown that cardiac mass, stroke volume, and exercise tolerance decrease over time in microgravity [3, 5]. Although these changes are generally reversible post-mission, they pose significant risks for astronaut health and performance, especially during re-entry or extravehicular activity.

Importantly, long-duration missions pose not only the challenge of altered gravity but also cumulative exposure to cosmic and electromagnetic radiation, which may, over time, compromise the functionality of organs and biological systems. These compounding stressors demand integrating approaches to both astronaut safety and terrestrial medicine.

With the prospect of deep-space exploration, including missions to Mars that may last up to 3 years, the physiological burden becomes even more pressing. Not only is the duration longer, but astronauts will be unable to rely on rapid medical evacuation. Preliminary data suggest that cardiovascular adaptation may differ by sex, potentially influenced by hormonal regulation of vascular tone and autonomic function [5]. Female astronauts, for instance, may exhibit greater susceptibility to orthostatic symptoms. Although the evidence is still emerging, these findings underscore the need for personalised approaches to pre-flight screening, in-flight monitoring, and countermeasure design.

The study of suborbital flight has produced new evidence that improves our knowledge about the effects of brief extreme gravitational changes on neuroendocrine and immunological homeostasis. Bosco et al. [6] found that short suborbital missions resulted in measurable decreases in plasma dopamine levels. These missions were also associated with increased cortisol and brain-derived neurotrophic factor levels, alongside systemic pro-oxidative and pro-inflammatory changes [6]. The research supports the conclusion that brief gravitational changes during minutes to hours create substantial stress for autonomic regulation and neurovascular integrity. The observed neurochemical changes may contribute to the acute fatigue symptoms, mood changes, and executive function deficits that astronauts experience after short flights. The study demonstrates that space mission planning and rehabilitation protocols need to consider both mechanical and cardiovascular stressors, neuroendocrine resilience, and oxidative balance. The research provides new opportunities to study autonomic dysfunction and stress hypersensitivity, and inflammation in Earth-based conditions.

Exercise remains the cornerstone of cardiovascular protection in space. On the ISS, astronauts engage in a regimented program that includes 60 min of aerobic training and 40–60 min of resistance training daily [7, 8]. These routines mimic the physiological demands of terrestrial activity, helping to maintain cardiac output, vascular compliance, and skeletal muscle mass. The parallels to exercise recommendations for Earth-bound populations are clear. Guidelines for cardiovascular disease prevention advocate at least 150 min of moderate aerobic activity and 75 min of strength-based exercise per week, yet many adults fail to meet these targets [9]. In contrast, astronauts must meet them simply to maintain baseline function.

Beyond exercise, technological innovations have begun to reshape in-flight countermeasures. One such development is the Gravity Loading Countermeasure Skinsuit (GLCS), which mimics the compressive and axial loading effects of gravity by applying tension from the shoulders to the feet. The GLCS has demonstrated potential in reducing spinal elongation, supporting posture, and preserving cardiovascular tone [10]. Importantly, it may also have applications for patients on Earth, particularly those suffering from orthostatic intolerance, chronic fatigue, or deconditioning from prolonged bed rest. Compression garments, resistance wearables, and simulated loading systems inspired by the GLCS are now being tested in rehabilitation and geriatric medicine.

The relevance of these countermeasures to terrestrial healthcare is growing. Many patients recovering from intensive care, cardiac surgery, or prolonged immobilisation experience the same core issues: reduced preload, impaired baroreflex sensitivity, and skeletal muscle wasting. The exercise regimens used in space have informed cardiac rehabilitation protocols on Earth, particularly for those with chronic heart failure or frailty syndromes. These overlaps suggest that space medicine can serve as a testbed for interventions that can be directly translated into clinical care.

Looking ahead, the future of cardiovascular protection in space will likely centre around adaptability and personalisation. Research into artificial gravity, including short-radius centrifuges, is ongoing. These devices could provide intermittent gravitational loading during long-duration missions, better preserving cardiovascular and vestibular function. Simultaneously, the rise of wearable biosensors promises to revolutionise in-flight monitoring. Real-time tracking of electrocardiogram, blood pressure, hydration status, and heart rate variability can allow astronauts and patients alike to receive tailored interventions on the basis of dynamic physiological data [7].

Further still, the integration of systems biology may usher in an era of precision countermeasures. Genomic and proteomic profiling could help identify astronauts at higher risk of cardiovascular deconditioning, informing bespoke exercise or pharmacological regimens. These same approaches are already gaining traction in cardiovascular care on Earth, from hypertension management to heart failure phenotyping. In this way, the convergence of aerospace medicine and precision health holds promise not only for space travellers but also for patients in hospitals, clinics, and homes around the world.

In conclusion, the cardiovascular system’s adaptation to microgravity is more than an academic curiosity; it is a compelling example of how human physiology responds to extreme environmental conditions. Through rigorous observation and innovation, space agencies have developed countermeasures that preserve health in the most unnatural of settings. As we prepare for missions to the Moon, Mars, and beyond, the imperative to protect cardiovascular function will only intensify. Yet the greatest value of this research may be what it teaches us about healing on Earth. In a world facing rising chronic diseases, aging populations, and growing demands for rehabilitation, spaceflight offers a template for resilience and a vision of medicine without boundaries.

## Data Availability

Not applicable.

## Abbreviations

GLCS: Gravity Loading Countermeasure Skinsuit
ISS: International space station
